# Influence Of Fear Of Falling And Built Environment On Physical Activity Among Diverse Older Adults with Cancer

**DOI:** 10.1093/geroni/igaf122.3795

**Published:** 2025-12-31

**Authors:** Kristen Fessele, Justin O’Leary, Anita Yafeh Mfuh, Jaime Gilliland

**Affiliations:** Memorial Sloan Kettering Cancer Center, Bridgewater, New Jersey, United States; Memorial Sloan Kettering Cancer Center, New York, New York, United States; Memorial Sloan Kettering Cancer Center, New York, New York, United States; Memorial Sloan Kettering Cancer Center, New York, New York, United States

## Abstract

Motivation for older adults with cancer (OAC) to engage in physical activity (PA) during and after cancer treatment may be limited by many factors. OAC may be affected by persistent symptoms limiting PA capability such as fatigue, pain and dyspnea, or lack safe home or neighborhood spaces to engage in activity. Fear of falling (FOF) is a major concern among OAC and may decrease PA and life-space mobility. To understand how OAC perceptions of cancer treatment, FOF and their local built environment (BE) influence PA behaviors, we utilized ResearchMatch.org to recruit a sample of 19 people with a history of cancer aged 65+ who were diverse in self-identified race, ethnicity, gender and sexual orientation. We developed an interview guide using Michie’s COM-B model (Capability, Opportunity, Motivation – Behavior) and conducted semi-structured interviews ranging from 24 to 60 minutes. Thematic Content Analysis was conducted by two investigators in NVivo v14. Critical themes included 1) regaining past PA levels vs. adjusting to new limitations; 2) high FOF levels may limit PA selection, regardless of fall history; 3) avoiding socialization and PA in public due to post-surgical changes in appearance; 4) home and local neighborhood layout impact opportunity to access safe spaces for PA and 5) desire to receive customized PA and health promotion education near end of treatment instead of while overwhelmed near diagnosis. Factors influencing motivation for PA in OAC are complex; clinicians should assess FOF and local BE to better customize PA recommendations that address these concerns and limitations.

